# Tumor mRNA-lipid nanoparticles via chimeric nanobody-lipid co-assembly

**DOI:** 10.7150/thno.123633

**Published:** 2026-01-01

**Authors:** Chuandong Zhu, Jing Wang, Md. Mofizur Rahman, Yun Zhang, Lixue Wang, Yuan Wan

**Affiliations:** 1 Department of Oncology, The Second Hospital of Nanjing, Nanjing University of Chinese Medicine, Nanjing, Jiangsu, China.; 2 Department of Hematology, The Affiliated Drum Tower Hospital of Nanjing University Medical School, Nanjing, Jiangsu, China.; 3 The Pq Laboratory of BiomeDx/Rx, Department of Biomedical Engineering, Binghamton University, Binghamton, NY, USA.; 4 Nanjing Regenecore Biotech Co. Ltd., Nanjing, Jiangsu, China.

**Keywords:** mRNA vaccine, HER2, nanobody, lipid nanoparticle, cancer

## Abstract

**Background:** Targeted mRNA-lipid nanoparticles (LNP) show great potential for cancer immunotherapy by delivering neoantigen-encoding messages to tumor cells, prompting immune responses against tumors. However, the challenges of inefficient production of targeting ligand-grafted LNPs and the immunogenic effects of polyethylene glycol (PEG) hinder their therapeutic effectiveness.

**Methods:** We introduced a simplified, one-step technique for creating PEG-free, human epidermal growth factor receptor 2 (HER2)-targeted mRNA-LNPs. This method incorporates self-assembled palmitoylated nanobodies (pNB), lipids, and mRNA that encode spike proteins (SP). We engineered cells to produce pNB, which were then mixed with lipids and mRNA at various ratios. Through hydrophobic interactions between the lipid tails and the palmitoyl groups, we assembled tumor-targeting mRNA-LNPs. We optimized both the lipid components and the quantity of pNB, and determined the optimal formulation based on a series of physicochemical characterizations of the LNP as well as in vitro cel assays. Building on this, we further conducted in vitro cytotoxicity assays targeting SP-expressing cells, followed by in vivo immune killing experiments.

**Results:** In vitro, SP-expressing tumor cells triggered strong immune responses and effective tumor cell destruction. Additionally, these pNB-LNPs demonstrated improved tumor-specific delivery, extended tumor retention, and considerable tumor volume reduction in vivo.

**Conclusion:** This streamlined, PEG-free LNPs platform that utilizes pre-existing immunity presents a flexible strategy for targeted cancer immunotherapy and applications in infectious diseases.

## Introduction

mRNA vaccines have emerged as a groundbreaking technology during the COVID-19 pandemic 1. These vaccines function by delivering synthetic mRNA that encodes the SARS-CoV-2 spike protein (SP) into cells, thereby prompting immune responses. Their rapid development, high efficacy, and adaptability have inspired research into their application in cancer treatment 2. Currently, mRNA-based cancer vaccines aim to stimulate immune recognition of tumor-associated antigens 2. However, their effectiveness continues to be limited due to weak tumor antigen immunogenicity and tumor evolution 3. Over the past four decades, the suboptimal outcomes of tumor antigen-targeting cancer vaccines underscore these challenges 3. An alternative strategy involves inducing tumor cells to express selected neoantigens that can elicit specific immune killing. This approach has been explored through the use of DNA plasmids, viruses, and microbes 4-7. While this strategy is promising, the delivery methods present practical hurdles. Intratumoral injection is not universally applicable, and intramuscular injections fail to achieve targeted neoantigen expression in tumor cells. To address these limitations, tumor-targeting mRNA vaccines for intravenous administration have been developed through the incorporation of targeting moieties into lipid nanoparticles (LNP) 8-12.

These tumor-targeting mRNA-LNP can specifically instruct tumor cells to express selected neoantigens, thereby promoting immune responses against tumors while reducing harm to healthy tissues 2, 13. However, the production of targeting ligand-grafted LNP is still not efficient 14, 15. Traditionally, targeting ligands are chemically conjugated to the LNP surface, a process that involves chemical group activation, ligand immobilization, and capping of unreacted chemical groups. The existing multi-step process can compromise ligand stability, cause payload leakage, and result in product loss 16. Additionally, using polyethylene glycol (PEG) to enhance LNP stability may trigger immunogenicity 17. Repeated exposure to PEG can trigger the formation of anti-PEG antibodies, leading to the rapid clearance of LNPs and hypersensitivity reactions.

**Figure 1:**
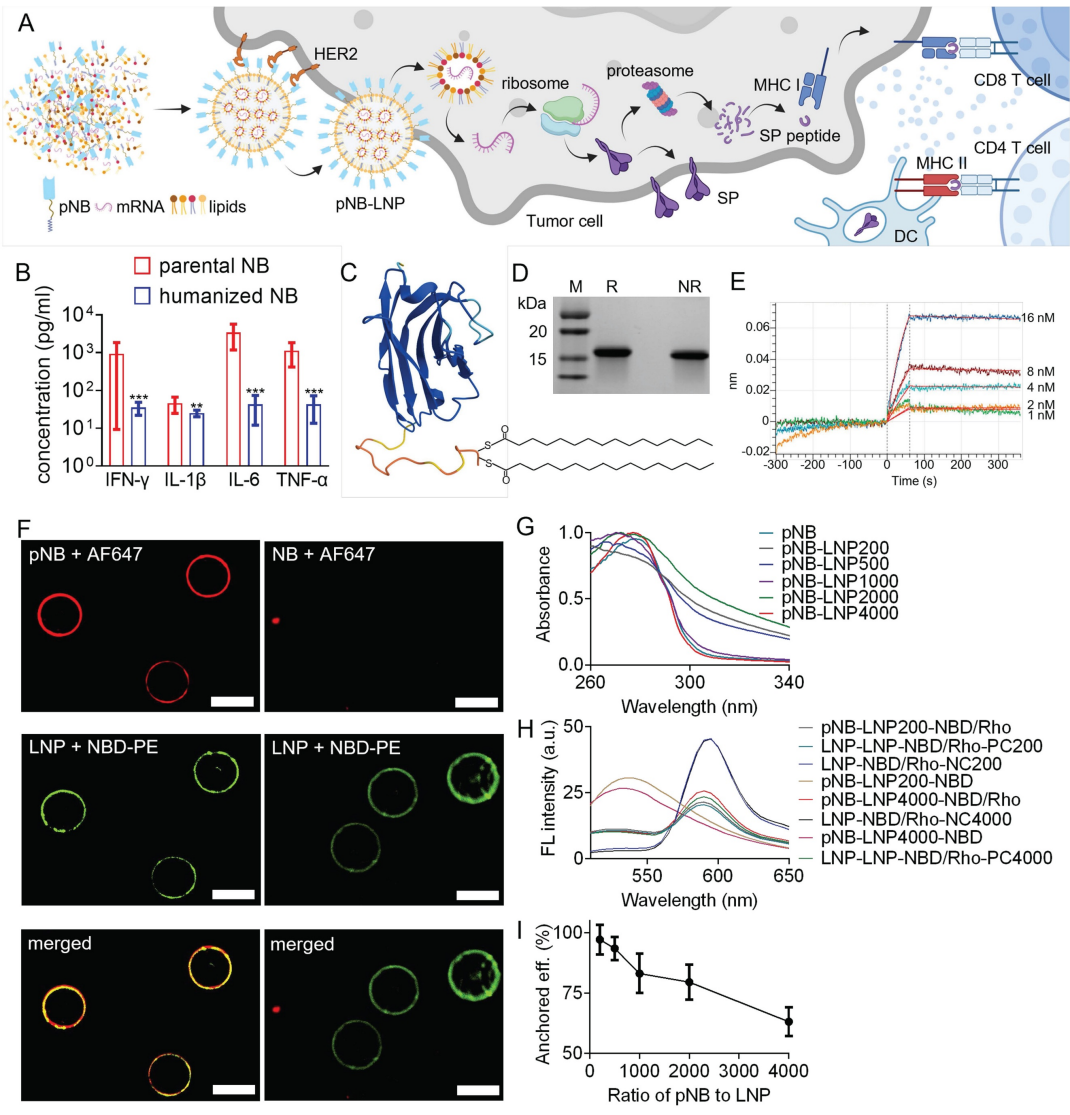
*Schematic representation of HER2-targeting mRNA-LNPs and characterizations of pNB.* (A) Flowchart illustrating self-assembled HER2-targeting mRNA LNP binding to HER2-overexpressing tumor cells, delivering mRNA into the cytosol, inducing SP expression, and triggering CD3^+^ T cell-mediated immune responses. (B) Cytokine release analysis of parental and humanized NB. Each data represents the Mean±SD of twelve replicates: *p < 0.05, **p < 0.01, and ***p < 0.001. (C) Structural elucidation of pNB. (D) SDS-PAGE gel analysis of CHO cell-produced pNB under reducing (R) and non-reducing (NR) conditions. (E) Kinetic analysis of pNB. (F) Staining of pNB-grafted LNP using AF647 labeled anti-His antibody and NBD-PE labeled lipid. NB without a palmitic acid tail failed to integrate into lipid membranes. (G) FRET emission fluorescence signals of pNB-grafted LNP. (H) Fluorescence signals of FRET-pair labeled pNB-LNP and LNP. (I) Decoration efficiency of pNB as a function of pNB quantity.

In this study, we introduce an improved technique for creating epidermal growth factor receptor 2 (HER2)-targeting PEG-free mRNA-LNP through the self-assembly of palmitoylated nanobodies (pNB), lipids, and mRNA encoding SP. Through electrostatic adsorption between mRNA and ionizable cationic lipids, followed by self-assembly with helper lipids, cholesterol, and the palmitoylated acid tail derived from pNB, non-lamellar tumor-targeting mRNA-LNP were prepared in one step. HER2 was selected as a target due to a combination of clinical and experimental advantages. HER2 is a well-established and overexpressed target for certain cancers, with a rich history of successful targeted treatments 18. It enables internalization of mRNA-LNP through receptor-mediated endocytosis, and in combination with the non-lamellar architecture, facilitates efficient cytosolic delivery of mRNA and endosomal escape. Moreover, as reported in our previous work, we successfully engineered an anti-HER2 nanobody (NB) capable of directing the self-assembly of immunoliposomes, with its robustness, reliability, and practical applicability thoroughly validated 19. The SP was chosen based on the premise that most individuals have been exposed to SARS-CoV-2 either through natural infection or vaccination. This widespread exposure confers pre-existing immunological memory, including SP-specific memory T and B cells. Here, we directly harnessed pre-existing immunity to test the immune recognition and clearance of tumor cells. The administration of these mRNA-LNP led to SP being presented on the surfaces of HER2-expressing tumor cells, resulting in targeted immune destruction (Figure 1A).

The key innovations of this study encompass three major aspects. First, the conventional PEG-dependent ligand conjugation strategy is replaced with direct insertion of pNB, enabling high-density ligand display on LNP without the need for multistep chemical modification. Second, the helper lipid composition was tuned to favor the formation of non-lamellar LNP architectures. Third, this platform leverages pre-existing immunity against non-tumor antigens, such as SP, to potentiate immune recognition and clearance of tumor cells, offering a conceptually distinct approach to tumor immunotherapy. In brief, this streamlined manufacturing method for tumor-targeting mRNA-LNP, combined with the use of pre-existing immune memory, holds promise for advancing tumor immunotherapy.

## Results

### Characterization of pNB

We humanized the anti-HER2 nanobody identified in our previous study to improve its biocompatibility 19. Humanized NBs significantly reduced IFN-γ, IL-6, and TNF-α release* in vitro*, by approximately 15 to 50 times (Figure 1B). Notably, this NB functions solely as a targeting ligand without inhibiting HER2 dimerization or affecting HER2-expressing tumor cells 19. The engineered pNB features the humanized NB, a flexible (G4S)_4_ linker, and a palmitic tail, arranged in an N- to C-terminal order (Figure 1C). The pNB yield is over 250-fold higher than that of chimeric nanobodies (cNB) containing an α-helix single transmembrane peptide 19. Approximately 11.7 mg of pNB with a purity of 96% can be harvested from a 40-ml supernatant (Figure 1D and Figure S1). Palmitoylation at two consecutive cysteine residues increased the molecular weight of NB by ~479 Da. In contrast, under an equivalent volume of supernatant, only ~44.8 µg of cNB with 95% purity were harvested. The *K_d_* of the pNB was 1.19 nM (Figure 1E), comparable to the non-engineered NB with a *K_d_* of 0.53 nM. Our results indicated that the ~7 nm neural linker and the ~2.4 nm palmitic tail did not interfere with the NB's binding affinity.

### Characterization of pNB-decorated LNP *in vitro*

Our pNB can spontaneously integrate into lipid membranes via its palmitic tail, as visualized by anti-His fluorescent antibodies, while NB without a palmitic tail failed to assemble with the membranes (Figure 1F). The presence of pNB on LNP (DSPC/DOPE/cholesterol) membranes was verified. UV-vis spectroscopy data indicated that increased pNB availability led to greater membrane integration (Figure 1G). Fluorescence resonance energy transfer (FRET) data revealed that pNB was homogeneously distributed over the membranes, independent of its quantity (Figure 1H). We aimed for input pNB-to-LNP ratios of 200:1, 500:1, 1000:1, 2000:1, and 4000:1, corresponding to the number of pNB per LNP. The experimentally determined ratios were 194:1, 467:1, 832:1, 1592:1, and 2526:1, respectively. The anchor efficiency of pNB decreased from 97.1% to 63.2% as the mixing ratio of pNB to LNP increased from 200 to 4,000 (Figure 1I). Equivalently, more than ~200 pNB were integrated into a ~70 nm LNP. In contrast, recent studies either omitted reporting the number of grafted antibodies or reported that only 2-7 antibodies were conjugated to each PEGylated LNP (LNP-PEG) 14, 15.

**Figure 2:**
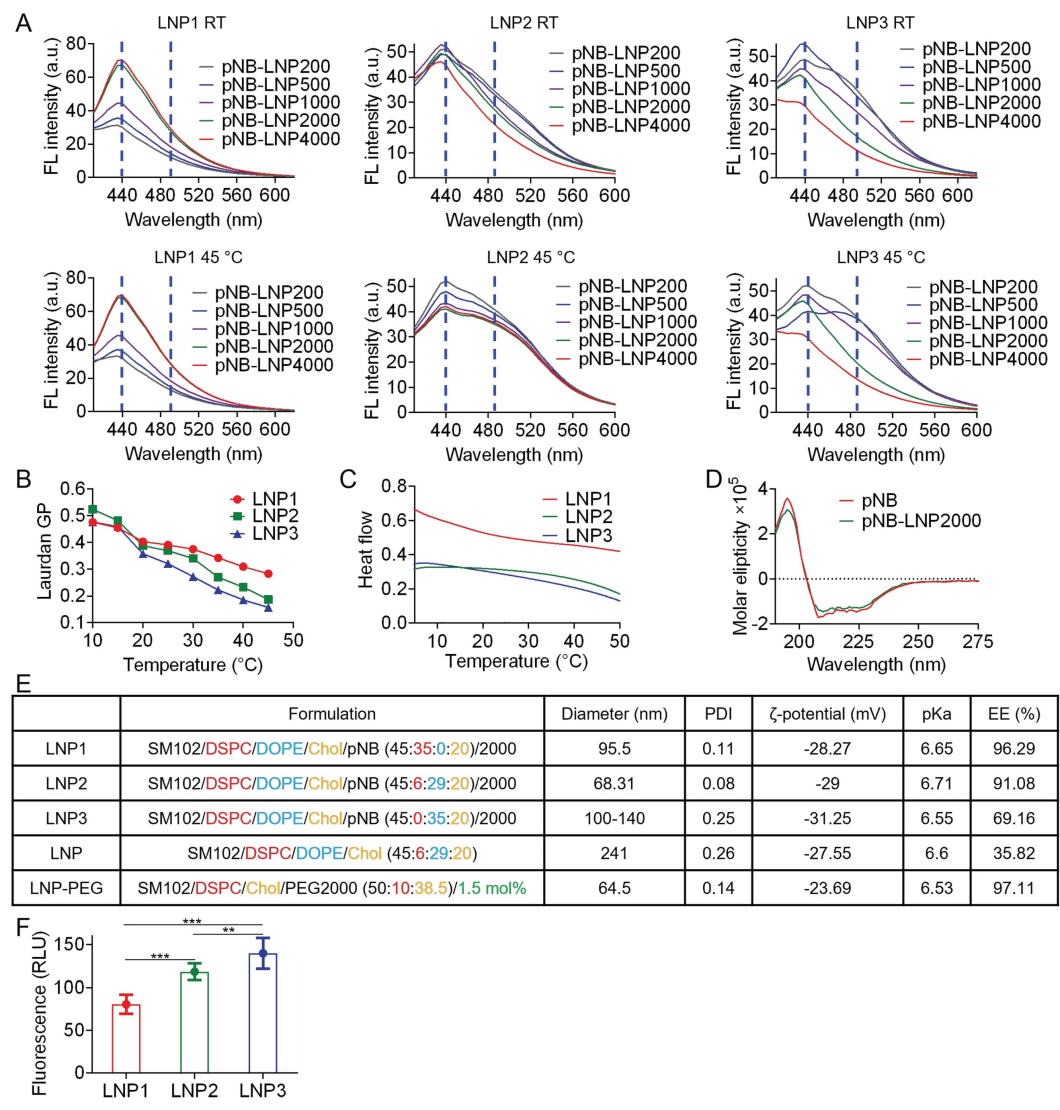
*Characterization of three pNB-LNP formulations.* (A) Fluorescence signals of Laurdan emission from different pNB-LNP formulations at room temperature (RT) and 45 °C. (B) Averaged ΔGeneralized polarization values as a function of temperature for various pNB-LNP^2000^ formulations. (C) Differential scanning calorimetry analysis of various pNB-LNP^2000^ formulations across different temperatures. (D) Circular dichroism spectra of pNB and pNB-LNP^2000^ in PBS. (E) Quantitative analysis of pNB-LNP^2000^ formulations, including diameter, polydispersity index (PDI), ζ-potential, pK_a_, and encapsulation efficiency (EE). (F) Cellular delivery efficiency of Cy5-modified mRNA using different pNB-LNP^2000^ formulations. Each data represents the Mean±SD of five replicates: **p < 0.01 and ***p < 0.001.

Saturated DSPC, cholesterol, and pNB can synergistically stabilize LNP 19, while unsaturated DOPE primarily promotes membrane fusion 20. Therefore, we further assessed the impact of pNB amounts and DSPC/DOPE ratios on LNP stability. LNP containing 6-35% DSPC remained stable at 45 °C, whereas DSPC-deficient LNP with lower pNB exhibited increased membrane fluidity, as indicated by a redshift in the emission peak (Figure 2A). Based on this finding and pNB anchoring efficiency, we concluded that 2,000 pNB was optimal for vaccine preparation. In this scenario, approximately 1,500 pNB were integrated into each LNP. This sufficient quantity of pNB could form a protein corona, effectively preventing pNB-LNP aggregation. Additional characterizations confirmed the thermostability of pNB-LNP and the absence of pNB micelles (Figure 2B-D). RiboGreen assays revealed that pNB-grafted LNP formulated with 6-35% DSPC achieved 91-96% mRNA encapsulation efficiency at a SM102/mRNA weight ratio of 10:1, comparable to LNP-PEG (Figure 2E). Of note, the reference LNP-PEG formulation contained 1.5 mol% PEG, calculated based on the total lipid content. Comparatively, increasing DOPE content enhanced the cell delivery of Cy5-modified mRNA (Figure 2F).

**Figure 3:**
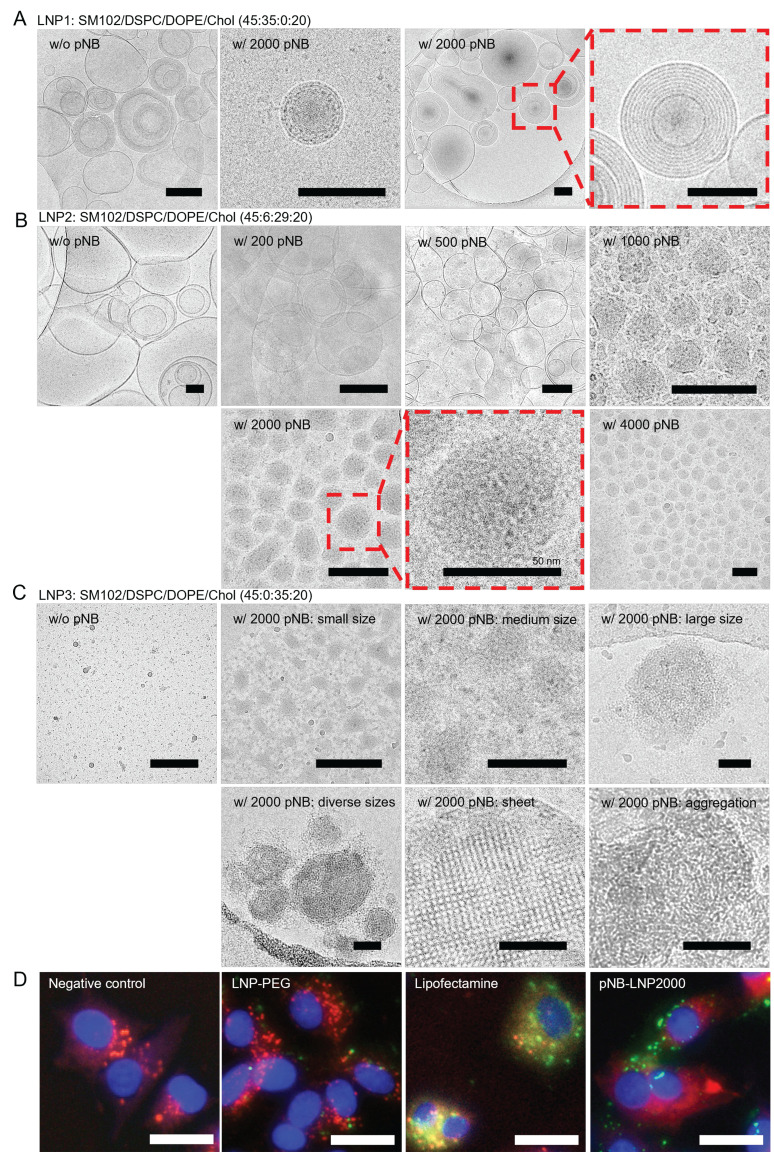
*Cryo-TEM images of various pNB-LNP.* (A) Typical multilamellar structures observed in LNP1. (B) Non-lamellar structures observed in LNP2 decorated with 200 or more pNB. (C) Clusters and sheet structures observed in LNP3. Scale bar: 100 nm. (D) Fluorescence images of LNP-cell interaction (Green: Alexa Fluor 488, Red: Lysoview 594, and Blue: DAPI). Scale bar: 20 µm.

LNP morphology was visualized under an electron microscope (Figure 3A-C). A 6:29 DSPC/DOPE ratio yielded LNP2 with non-lamellar structures, which may consist of a mixture of disordered hexagonal, minor lamellar, and even inverse micellar phases (Figure 3B). Comparatively, omitting DOPE directly led to the formation of multilamellar structures in rigid LNP1 (Figure 3A), and absence of DSPC may result in the aggregation of flexible LNP3 or fusion into sheet-like configurations (Figure 3C). The structural transformation might arise from the balance of DSPC/DOPE/cholesterol and curvature frustration induced by the palmitic tail 21-23. Asymmetrically shaped DOPE and SM102 inherently promote non-lamellar structures 24. The palmitic tail further promotes non-lamellar phase formation by inducing membrane curvature through increased lateral pressure within the membrane 25. Meanwhile, cholesterol stabilizes the high-curvature membranes and works with DSPC to enhance LNP stability 26. The optimized formulation achieved a balance between rigidity and flexibility, which successfully yielded non-lamellar structures in LNP2. Notably, non-lamellar LNPs are more effective at disrupting membranes and delivering mRNA to cytosol than lamellar LNPs 27. In contrast, in the absence of DSPC, pNB, or both, LNP formation was either unsuccessful or resulted in LNPs with uncontrolled size and aggregation. It is noteworthy that non-lamellar structures have been increasingly associated with membrane remodeling and enhanced endosomal escape in lipid-based delivery systems. Zheng *et al.* demonstrated that LNP topology can regulate cytosolic delivery independently of lipid composition 22, while Pattipeiluhu *et al.* reported that inverted liquid-crystalline phases significantly improve siRNA transfection efficiency compared to conventional lamellar structures 23. Reviews underscore the fusogenic properties of non-lamellar lipids and their emerging relevance in next-generation nanomedicine design 24. Foundational biophysical studies further established that lamellar-to-H_II phase transitions facilitate membrane fusion and destabilization 20, 28. Collectively, these findings suggest that the non-lamellar features in our pNB-LNP formulations may contribute to enhanced functional delivery. While there is growing evidence that non-lamellar architectures contribute to improved mRNA delivery efficiency in LNP systems, we recognize that delivery outcomes are governed by multiple interrelated factors, such as uptake pathways, and a direct causal relationship cannot be attributed solely to morphology. We further analyzed the intracellular trafficking of LNP-PEG, pNB-LNP2000, and lipofectamine (LF) in SK-BR-3 cells (Figure 3D). Colocalization of green fluorescent from the mRNA with red fluorescence from the endosome was observed in LF group, indicating that the LF entered endosomal compartments, with a subset successfully escaping from lysosomes into cytosol. By contrast, the LNP-PEG demonstrated limited internalization, which can be attributed to electrostatic repulsion between the PEG layer and the cell membrane surface. In pNB-LNP group, substantial green fluorescence was observed outside of endosomal compartments, indicating effective endosomal escape and cytosolic delivery of mRNA. The finding partially supports the notion that non-lamellar structures can facilitate cytosolic delivery of mRNA.

**Figure 4:**
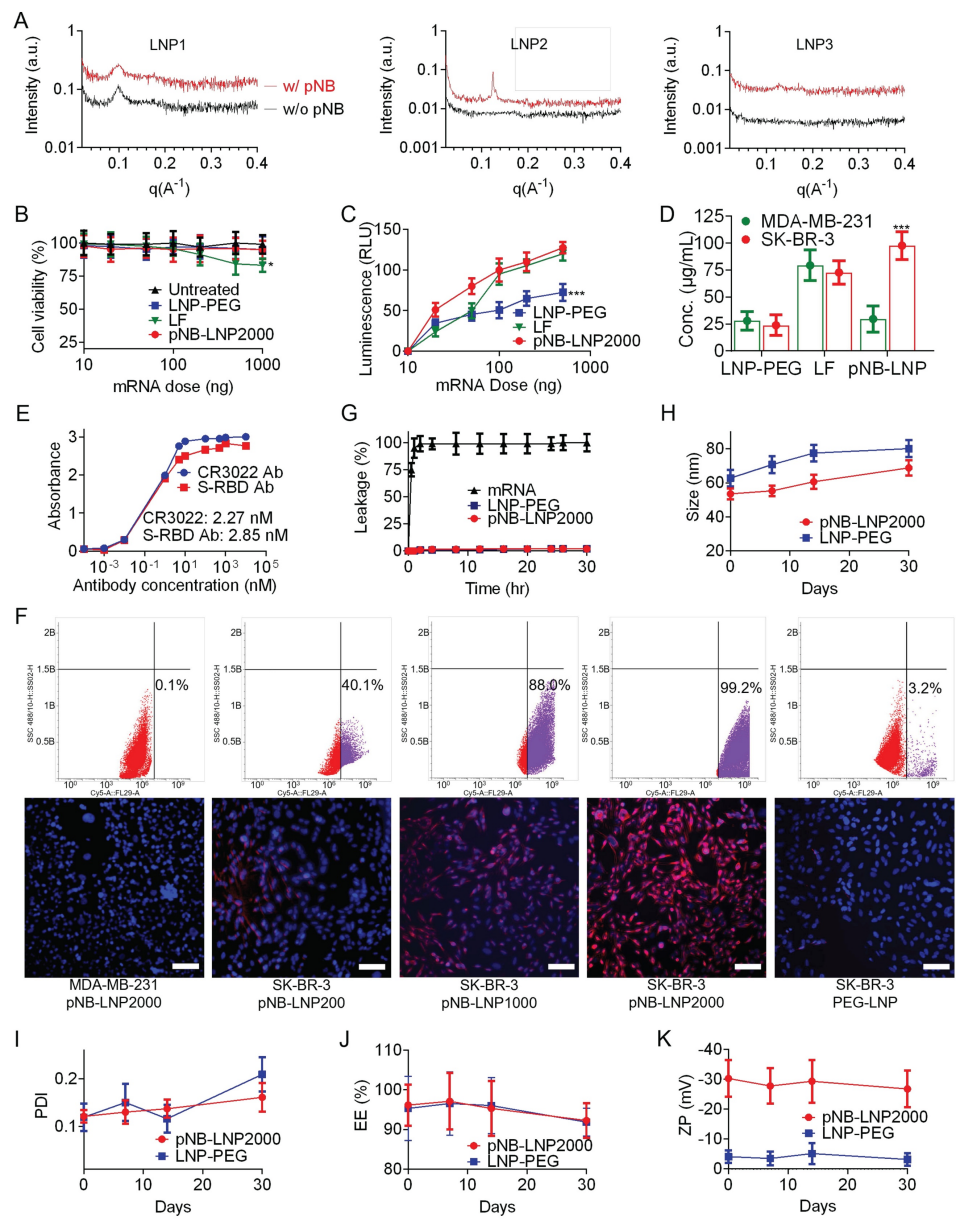
*Optimization of mRNA dose and assessment of pNB-LNP.* (A) SAXS scattering curves for different pNB-LNP formulations. (B-C) Cell viability and luminescence of SK-BR-3 cells treated with 10-1,000 ng mRNA using three different vaccines. (D) Quantitative data of SP expression in HER2-null MDA-MB-231 cells and HER2-overexpressing SK-BR-3 cells treated with three mRNA vaccine types. (E) Binding assessment of expressed SP using two commercially available antibodies. (F) Assessment of pNB-LNP internalization in MDA-MB-231 cells and SK-BR-3 cells using flow cytometry (upper) and fluorescence microscopy (lower). pNB-LNP were stained with AF647-labeled anti-His antibodies. (G) 30-hour mRNA leakage profiles across three groups. (H-K) Changes in size, PDI, EE, cargo leakage, and ζ-potential of pNB-LNP and LNP-PEG stored at 4 °C in PBS for up to 30 days. Each data represents the Mean±SD of five replicates: *p < 0.05 and ***p < 0.001.

The small-angle X-ray scattering (SAXS) data corroborated the TEM findings and indicated the integration of pNB (Figure 4A). It was challenging to differentiate the pNB signal within both rigid LNP1 and flexible LNP3. Higher DSPC content generally favored less curved, more lamellar structures. For example, liposomes typically exhibit lamellar structures due to their relatively high PC content (~30-70%). Mammalian cell membranes in a lamellar structure generally contain ~40-50% PC. In contrast, LNP, which generally contain ~10% PC along with cone-shaped lipids such as DOPE, often consist of a mixture of lamellar, inverse hexagonal (H_II_), and cubic phases. Our SAXS data revealed lamellar Bragg peaks at higher DSPC content (LNP1).

Conversely, increasing the proportion of DOPE (LNP2) resulted in the disappearance of lamellar peaks and the emergence of H_II_ reflections, indicating formation of inverse hexagonal structures. More specifically, DSPC with its cylindrical shape stabilizes lamellar bilayers, whereas DOPE with its small headgroup and bulky acyl chains promote negative curvature and H_II_ phase formation. Without DSPC (LNP3), only hexagonal and cubic phases were observed, suggesting uncontrolled assembly, while a control without pNB showed no discernible structure. Our findings align with previous studies showing that unsaturated PE can drive lamellar to H_II_ transition, which saturated PC can stabilize lamellar structures 20, 22-24, 28-32. Therefore, our SAXS data support the conclusion that structural heterogeneity in pNB-LNP arises from the interplay between DSPC and DOPE. Of note, the detected signal intensity in SAXS is not an absolute quantitative measure. Direct comparison of scattering intensity between different samples is generally not valid. SAXS primarily focuses on the shape and features of the scattering profile, rather than on absolute intensity values.

Collectively, LNP2 (pNB-LNP^2000^) was used in the following studies. Subsequently, we optimized the mRNA dosage and analyzed SP expression. Optimal expression was obtained with 100-200 ng of luciferase mRNA housed in pNB-LNP^2000^, LF, and LNP-PEG. An excessive dosage of mRNA did not substantially boost the luciferin signal but could jeopardize cell viability due to excessive translation (Figure 4B). pNB-LNP^2000^ demonstrated a greater signal intensity compared to LNP-PEG owing to its active targeting (Figure 4C). LF achieved efficient *in vitro* transfection by leveraging its positively charged surface to non-specifically adsorb onto cell membranes via electrostatic interactions. Therefore, we selected 100 ng of mRNA for further analysis. After transfecting HER2-overexpressing SK-BR-3 cells, the SP concentration in the pNB-LNP^2000^ group cell lysates was approximately 98 µg/ml, which is over four times higher than that in the LNP-PEG group (Figure 4D). Conversely, when HER2-null MDA-MB-231 cells were transfected, SP concentrations in both the pNB-LNP^2000^ and LNP-PEG groups were comparable, and they were less than a third of the concentration found in the LF group. The expressed SP was detected by two commercial anti-SP antibodies, and the EC_50_ values obtained were comparable to reported data (Figure 4E). Flow cytometry and an immunofluorescence assay showed that increased pNB surface exposure enhanced the uptake of pNB-LNP in SK-BR-3 cells, ultimately leading to elevated SP expression on SK-BR-3 cell membrane surface (Figure 4F). In contrast, pNB-LNPs were not efficiently internalized by HER2-null MDA-MB-231 cells. Similarly, PEG-LNP lacking pNB exhibited negligible internalization by SK-BR-3 cells, likely due to repulsion by the cell membrane resulting from its strong negative surface charge. Both pNB-LNP^2000^ and LNP-PEG maintained mRNA at room temperature for over 30 hours with minimal leakage (Figure 4G). In addition, we investigated the physicochemical evolution of our pNB-LNP^2000^ across 30 days at 4 °C. No significant alterations in size, mRNA content, or surface charge were observed, indicating pNB-LNP could be preserved for at least 30 days *in vitro* (Figure 4H-K).

### Anti-SP-mediated immune responses *in vitro* and *in vivo*

**Figure 5:**
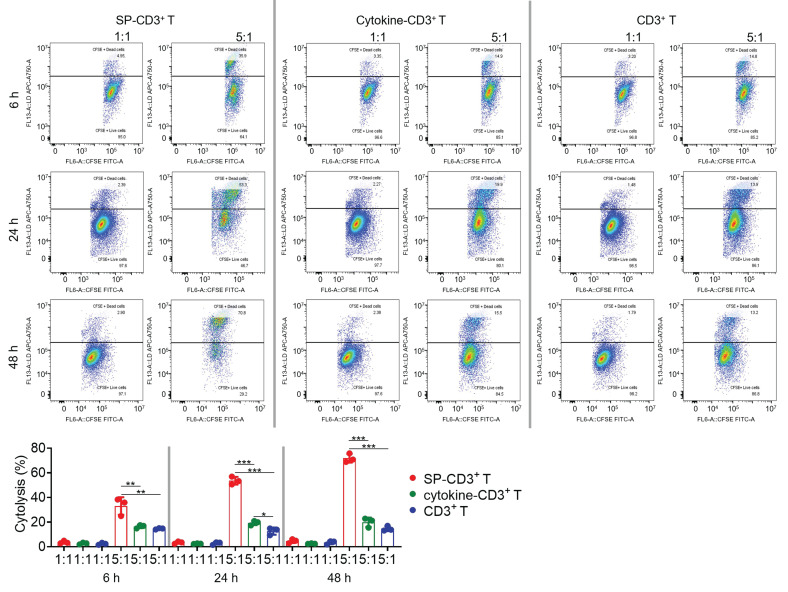
*T cell-mediated immune killing of SP-expressing HEK293 cells.* SP- and cytokine-stimulated T cells, along with unstimulated counterparts, were incubated with target cells in 1:1 and 5:1 ratio for 6, 24, and 48 hours. Cytotoxicity data were further quantified. Each data represents the Mean±SD of three replicates: *p < 0.05, **p < 0.01, and ***p < 0.001.

Endogenously SP-overexpressing HEK293T cells were utilized to assess *in vitro* immune killing (Figure S2A). Since the commercial anti-SP hIgG1 antibody (CR3022) failed to elicit antibody-dependent cellular cytotoxicity with Jurkat cells or fresh peripheral blood mononuclear cells (Figure S2B), antibody-dependent cellular phagocytosis and complement-dependent cytotoxicity were not investigated. Instead, we directly assessed cellular immune killing. We optimized the recombinant SP dosage, testing both 12.5 µg/mL and 25 µg/mL. For comparison, control groups received either commercial combined cytokines or plain cell culture medium. Using flow cytometry to monitor markers for both immature and mature DCs, including CD14, CD40, CD80, CD83, and CD86, we found that 25 µg/mL of SP was more effective at developing SP-loaded dendritic cells (DC) (Figure S2C-D). For subsequent experiments, SP-loaded DCs were prepared using 25 µg/mL of SP, and the expression levels of relevant markers were re-confirmed to meet established criteria. Microscopic examination revealed that DCs treated with 25 µg/mL SP underwent a morphological transformation from a typically round or oval shape to irregular forms characterized by elongated dendritic structures (Figure S3). Next, SP-loaded DCs were used to activate CD3^+^ T cells at a 1:10 ratio. Activated SP-CD3^+^ T cells killed SP-expressing HEK293T cells at an effector-to-target (E:T) ratio of 5:1, demonstrating increasing killing efficiency over time (Figure 5). Following 48 hours of co-incubation, approximately 71% cytotoxicity was observed. In contrast, cytokine-CD3^+^ T and naïve T cells displayed limited immune killing activity with cytotoxicity less than ~20%.

**Figure 6:**
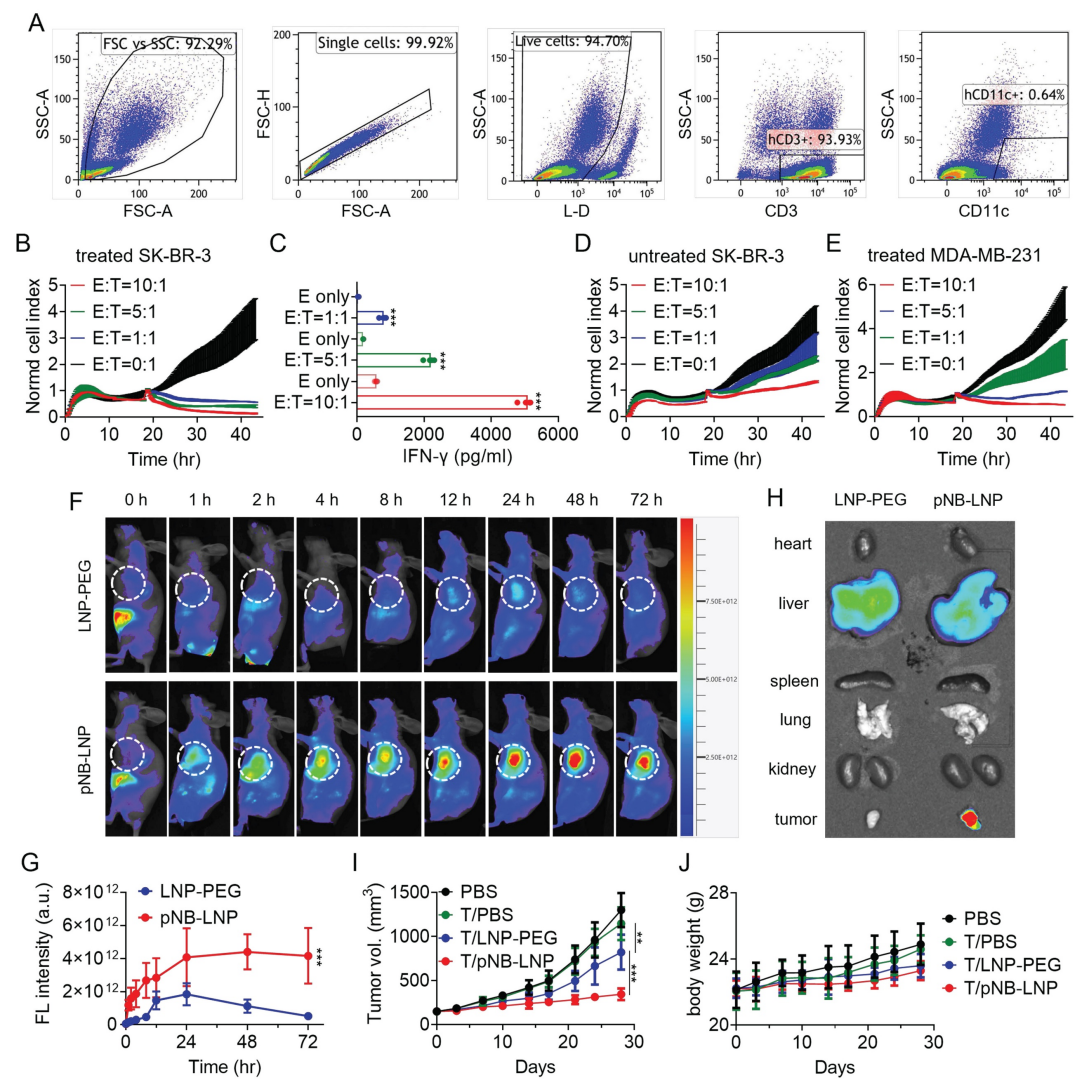
*Characterization of CD3^+^ T cells and cytotoxicity against cancer cells.* (A) Characterization of CD3^+^ T cells following co-incubation with SP-loaded DCs. (B) Cytotoxicity of CD3^+^ T cells co-cultured with pNB-LNP treated SK-BR-3 cells at different E:T ratios over 48 h. (C) IFN-γ in the supernatant released from activated CD3^+^ T cells in response to pNB-LNP^2000^ treated SK-BR-3 cells. (D) Normalized cell index of activated CD3^+^ T cells in response to SK-BR-3 cells without pNB-LNP^2000^ treatment. (E) Normalized cell index of activated CD3^+^ T cells in response to pNB-LNP^2000^ treated MDA-MB-231 cells. (F) *In vivo* fluorescence images of tumor-bearing mice after intravenous injection of the two formulations. (G) Quantitative data of fluorescence intensity in the tumor for the two groups over 72 hours. (H) Ex vivo fluorescence signals in main organs and tumor in the two groups. (I) Tumor volume of SK-BR-3 tumor xenograft in mice after mRNA vaccine or placebo administration. (J) Average body weight of mice in each group. Each data represents the Mean ± SD of three replicates: **p < 0.01 and ***p < 0.001.

Having established the effective killing of SP-expressing HEK293T cells by SP-CD3+ T cells, we then proceeded to evaluate their capacity to eliminate pNB-LNP^2000^-treated cancer cells. Real-time cell analysis was chosen, given flow cytometry's inherent operational complexity, including pre-labeling. Continuous and label-free monitoring of immune killing was conducted over 24 hours. Flow cytometry analysis indicated that the cells responsible for the immune-mediated killing were predominantly CD3⁺ T cells. (Figure 6A). The results showed that activated SP-CD3^+^ T cells effectively killed pNB-LNP^2000^ treated SK-BR-3 cells, with a positive correlation between killing efficiency and the E:T ratio (Figure 6B). Consistent with this, supernatant interferon-γ levels also increased with higher E:T ratios (Figure 6C). In contrast, no significant killing effect was noted for untreated SK-BR-3 cells (Figure 6D). A very limited cytotoxic effect of SP-CD3^+^ T cells on pNB-LNP^2000^-treated MDA-MB-231 cells was observed only at an E:T ratio of 10:1 (Figure 6E). Next, we investigated whether pNB-LNP^2000^ could induce anti-tumor immune responses in a mouse tumor model. The biodistribution of pNB-LNP^2000^ and LNP-PEG was assessed with SK-BR-3 xenografted mice. pNB-LNP^2000^ accumulated in the tumor for over 72 hours, exhibiting higher fluorescence intensity compared to LNP-PEG (Figure 6F). At 72-h timepoint, signal intensity of pNB-LNP^2000^ was ~8-fold higher, confirming the active targeting of pNB (Figure 6G). In contrast, LNP-PEG, relying on passive targeting, accumulated in the tumor for 24 hours before diminishing. *Ex vivo* tissue imaging further confirmed that pNB-LNP^2000^ accumulated in tumor more efficiently than LNP-PEG (Figure 6H). To determine therapeutic efficacy, NOG mice were intravenously injected with 1×10^7^ SP-CD3^+^ T cells or an equivalent volume of saline. LNP-PEG and pNB-LNP^2000^ were administrated in six doses, repeated every five days. By day 28, the tumors in the PBS group reached a size of ~1,300 mm^3^. SP-CD3^+^ T cell administration alone decreased tumor volume to ~1,150 mm^3^, while no significant difference was found between the two PBS groups. Compared to the saline-treated group, tumor volumes in mice receiving LNP-PEG and pNB-LNP^2000^ decreased by 36.7% and 73.4%, respectively (Figure 6I). The body weight of the pNB-LNP^2000^ group decreased by ~10%, showing a statistically significant difference compared to the other three groups (Figure 6J). Significant immune killing can be identified in tumor tissue (Figure S4). Tumor sections from the pNB-LNP group exhibited a markedly lower number of Ki-67-positive cells compared to the control groups, along with noticeably weaker staining intensity, reflecting a reduced proliferative rate. Tunel staining indicated that activated CD3^+^ T cells induced massive apoptosis in tumor cells. We did not observe significant cytotoxicity in heart, spleen, lung and kidney. Owing to hepatic enrichment of both PEG-LNP and pNB-LNP, we noted mild morphological alterations in hepatocytes, more pronounced in the pNB-LNP group. Nevertheless, the adverse effects appeared limited, as no significant changes in body weight were observed.

## Discussion

While ligand-conjugated PEGylated LNP as mRNA vaccines have been shown promising immunotherapeutic potential in cancer, the field remains in its infancy, with relatively few studies reported to date. Anti-CD44 peptide-conjugated LNP has demonstrated enhanced tumor accumulation and anti-tumor efficacy by delivering siRNA 10. However, these LNP rely on PEG as a stabilizing component, which raises potential concerns regarding anti-PEG immune responses. Another study reported mRNA-LNP targeting both epidermal growth factor receptor (EGFR) and folate hydrolase 1 9, aiming to enhance mRNA delivery efficiency to tumors through dual targeting. Instead of chemically conjugating two antibodies to PEG, thereby bypassing direct chemical modification of the LNP surface, the researchers constructed bispecific antibodies. One domain recognizes PEG, and the other recognizes either EGFR or folate hydrolase 1. A potential limitation of this approach is the non-covalent nature of the interaction, which may lead to detachment of the bispecific antibodies from the LNP surface. Moreover, the study did not evaluate therapeutic efficacy. It only focused on demonstrating that dual-targeted delivery can increase mRNA delivery efficiency by several fold. In another study, the LNP themselves lacked inherent tumor-targeting capability but were used to deliver mRNA encoding a HER2-CD3-Fc bispecific antibody. This strategy enforced the expression of HER2 on tumor cells and leveraged the CD3-binding domain to recruit T cells, thereby enabling immune-mediated tumor cell killing 11. However, this strategy often requires the use of HLA-A*02:01-restricted peptides, such as E75 (HER2/neu 369-377) and GP2 (HER2/neu 654-662), to activate T cells, in order to enhance their ability to effectively kill tumor cells. DNA-LNP targeting HER2 and folate have also been developed to enhance LNP uptake by cancer cells 12. However, this study focused solely on demonstrating enhanced LNP uptake in zebrafish and did not assess immunotherapeutic efficacy. In short, current studies remain limited in terms of evaluating therapeutic efficacy. Many do not involve mRNA delivery at all, and among those that do, tumor treatment outcomes are not assessed. In contrast, our study achieved tumor-targeted delivery, successfully induced SP expression in tumor cells, and demonstrated effective immune-mediated killing using SP-activated CD3^+^ T cells.

In parallel, to address the immune responses associated with PEG, poly(carboxybetaine) (PCB) lipids have been explored as surrogates for PEG-lipids in mRNA formulations 33. Studies have shown that PCB-containing LNP can enhance mRNA transfection efficiency of peripheral T cells, exhibit a favorable immunotoxicity profile, and effectively mitigate the accelerated blood clearance commonly observed with PEGylated LNP. Of note, this study did not include active targeting function. In another study, high-density brush-shaped poly(ethylene glycol) methyl ether methacrylate lipids were used to prepare mRNA-LNP 34. This strategy enhances protein production through the reduction of anti-PEG antibody interactions, and comprehensively demonstrates the low immunogenicity of the polymer, which allow for the repeated administration of mRNA-LNP. This study primarily focused on evaluating immunological safety. It did not employ an active targeting strategy, nor did it demonstrate therapeutic efficacy.

As an alternative to polymer-based formulations, a recent strategy seeks to entirely eliminate polymers by forming a protein corona on the surface of LNP 35. Colleagues mixed selected lipids, cholesterol, and apolipoprotein E (ApoE) and prepared PEG-free LNP. ApoE-coated LNP can effectively bind to low-density lipoprotein (LDL) receptors in the liver within 20 minutes of administration, thereby facilitating mRNA delivery to hepatocytes while restricting extrahepatic distribution. Similarly, Apolipoprotein A1 (ApoA1) has also been used as a protein corona on LNP surface 36. The ApoA1-coated LNP successfully delivered siRNA to myeloid cells and their bone marrow progenitors. In addition, antisense oligos and mRNA can be loaded into ApoA1-LNP for other types of nucleic acid therapeutics. Recently, Evans blue-modified LNPs capable of recruiting albumins to their surface have been reported. Unlike direct conjugation of albumin onto the LNP surface, this strategy utilizes Evans blue to non-covalently recruit endogenous albumins. The adsorbed albumin serves as a biological camouflage to evade immune clearance, thereby enhancing mRNA delivery efficiency 37. In brief, these studies enable the formation of a protein corona on the LNP surface, but they do not address the issue of targeting non-hepatic tumors.

Conventional strategies for preparing tumor-targeted LNP, including those employing click chemistry, generally require a multi-step process comprising LNP surface functional group activation, purification, ligand conjugation with capping, and an additional purification step 38, 39. Even assuming a minimal loss of 10% at each stage, the overall yield typically does not exceed ~65%. Moreover, the entire procedure requires no less than 12 hours to complete. Another critical limitation of conventional approach is that only a small fraction of targeting ligands can be successfully conjugated to the LNP surface. By contrast, our pNB-LNP enables one-step self-assembly, followed by simple filtration to remove free protein, lipids, and unencapsulated mRNA. The ligand density on the LNP surface is tunable, and the approach is readily compatible with microfluidic manufacturing, thereby facilitating scalability.

Leveraging pre-existing immunity from common vaccines, such as the hepatitis B vaccine, or hyperacute rejection against xenogeneic antigens like porcine αGal antigen 4, 6, 7, tumor-targeting mRNA-LNP can guide tumor cells to express relevant pathogen antigens, thereby triggering specific immune destruction of tumor cells. In contrast to epigenetic drugs that promote systemic neoantigen expression and evoke extensive immune responses, tumor-targeting mRNA-LNP can reduce systemic side effects. Moreover, the PEG-free pNB-LNP might improve biocompatibility 17. Its reduced surface hydrophilicity and negative charges could enhance cellular engagement. The deduction needs to be further examined in the future. NB-mediated targeting and the non-lamellar lipid structure further facilitate mRNA delivery. Even without active targeting capability, palmitoylated protein- or peptide-modified LNP could be substituted for LNP-PEG. In addition to palmitoylation, myristoylation can be considered. Both enable proteins to readily integrate into lipid membranes without comprising protein biological functions 40.

Lastly, despite extensive research on lipid formulations and amphiphile self-assembly, the nanostructure of LNP, particularly the arrangement of lipids and nucleic acids, remains poorly understood. The introduction of lipidated proteins further complicates LNP structural investigations. While we demonstrated the formation of non-lamellar structures through optimized lipid and pNB formulations, elucidating the underlying mechanisms and developing improved formulations remain critical objectives.

## Conclusions

In conclusion, pNB-LNP mRNA vaccines effectively target cancer cells that overexpress HER2, promoting SP expression and leading to immune-mediated tumor destruction. These antigen-targeting mRNA vaccines utilize lipid/lipidated ligand-based self-assembly and show significant promise in treating infectious diseases, oncology, and immune modulation, potentially revolutionizing preventive and therapeutic medicine.

## Materials and Methods

For animal experiments, we followed National Institutes of Health ethical guidelines and we received approval from the Institutional Animal Care and Use Committee of the Model Animal Research Center at the Second Hospital of Nanjing (202303A049), Nanjing University of Chinese Medicine. No tumor volume exceeded the humane endpoint of 2,000 mm^3^.

### pNB production with HEK293T cells

Anti-HER2 NB was provided by Regenecore (Nanjing, China). Its screening process, amino acid sequence, and comprehensive characterization were reported in our previous study 19. Regenecore conducted the humanization process and characterization. The sequence of the humanized NB is available from either Regenecore or the authors upon reasonable request. Plasmids for pNB production were synthesized and supplied by Genscript. HEK293T cells were cultured in high-glucose DMEM containing 10% FBS and 100 U/ml penicillin, 100 μg/ml streptomycin (Invitrogen) in a humidified atmosphere at 37 °C and 5% CO_2_. Approximately 2 µg of plasmid DNA was mixed with Lipofectamine 3000 (Thermofisher) according to the manufacturer's protocol and added to HEK293T cells at ~80% confluence in a 6-well plate. After a 12-hour incubation, the medium was replaced with fresh DMEM, and the cells were further cultured for an additional 72 hours at 32 °C. Both secreted pNB from the culture supernatant and pNB from the cell lysate were harvested at the 72-hour time point. pNB purification was performed using Ni-NTA resin (Qiagen) via the His-tag affinity method, following the manufacturer's instructions. The eluates were subsequently concentrated using ultrafiltration (Millipore, UFC5010). Protein size was assessed by SDS-PAGE alongside a molecular weight marker (Bio-Rad, 1610374S). The obtained pNB was analyzed by SEC-HPLC (Arc Bio, GR21010434). The purified NB and pNB were examined by matrix-assisted laser desorption/ionization-time-of-flight-mass spectrometry (MALDI-TOF-MS).

### Affinity measurement

The binding kinetics of purified NB and pNB to HER2 were determined using the Octet RED 96e system. Experiments were performed at 30 °C and reagents were prepared in 0.1% BSA, 0.02% Tween20 PBS, pH 7.4 buffer. Human HER2 was immobilized onto biosensors followed by association and dissociation measurements with NB and pNB for a time window of 70 and 30 seconds, respectively. Data analysis was performed using the software provided by the instrument.

### Preparation of LNP

LNPs were formulated at a 10:1 weight ratio of ionizable lipid to mRNA. The ethanol phase, which comprised the cone-shaped ionizable lipid SM102, cholesterol, DOPE, and DSPC in varying molar ratios (see Supplementary Fig. 3f), was blended with an aqueous phase containing SARS-CoV-2 mRNA (OZ Biotechnology) and pNB in a 25 mM sodium acetate buffer (pH 4). LNPs were generated through microfluidic mixing at a 3:1 volume ratio and subsequently dialyzed overnight against 1xPBS utilizing dialysis cassettes with a 10 kDa molecular weight cutoff (Thermo Scientific). Following dialysis, the solution was filtered through a 300 kDa ultracentrifugation membrane and stored at 4 °C. The dimensions and polydispersity index (PDI) of the LNPs were evaluated using an NS-300 (Malvern Panalytical). Furthermore, the concentration of leaked mRNA was quantified employing a fluorescence detection kit following the manufacturer's instructions.

### RiboGreen assay

The encapsulation efficiency (EE%) of LNP formulations was evaluated using the Quant-iT™ RiboGreen assay (ThermoFisher, R11490) according to the manufacturer's instructions. In summary, LNPs were either treated with 0.5% w/v Triton X-100 (Sigma-Aldrich, T8787) to disrupt their structure and release mRNA, or they remained untreated. Both treated and untreated LNPs were diluted to below a concentration of 1 μg mRNA/mL and combined with an equal volume of RiboGreen assay solution (200-fold dilution). Standard curves were created using free mRNA solutions ranging from 0.1 to 1.0 μg mRNA/mL, both with and without 0.5% w/v Triton X-100. The concentrations of free and total mRNA in the formulations were determined by measuring bulk fluorescence and comparing the results to the standard curves. EE% was calculated as 
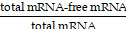
×100%.

### LNP pKa measurements

The pKa of each LNP was determined using the 6-(p-Toluidinyl)-2-naphthalenesulfonic acid (TNS) assay (Sigma-Aldrich, T9792). A buffer solution was prepared by combining 150 mM sodium chloride, 20 mM sodium phosphate, 25 mM sodium citrate, and 20 mM sodium acetate, with the pH adjusted in 0.5-unit increments from 2 to 12. In a 96-well plate, 140 µL of each pH-adjusted buffer solution and 5 µL of LNP were added in triplicate. TNS was then introduced to each well to a final concentration of 6 µM. Fluorescence measurements were recorded using a plate reader (Spark, Tecan).

### Zeta potential measurement

The zeta potential was measured utilizing a Zetasizer Nano ZS. A sample of 10 μL was diluted in 990 μL of deionized water and subsequently transferred to a Malvern Clear Zeta Potential cell. Three independent aliquots were analyzed, with three measurements conducted aliquot to ensure accuracy.

### Nanosight measurement

LNP and pNB-LNP^2000^ samples were suspended in 200 μl of PBS. Size distribution of LNP and pNB-LNP^2000^ were measured with Nanosight NS300 according to manufacturer's instructions followed by automated analysis with NTA software.

### Fluorescence microscope

pNB-LNP was prepared as previously described and stained with anti-His tag antibodies (Santa Cruz, AF647, 1:500) at 4 °C for 3 h, and LNPs were labeled with NBD-PE. Surplus antibodies and NBD-PE were removed with a centrifugal filter ultracel-300 membrane (Millipore, MRCF0R0300). The antibody-labeled pNB-LNP was collected and resuspended in PBS. Fluorescence images were taken with Olympus IX83. In pNB-LP^2000^/SK-BR-3 cell binding assay, ~1×10^4^ SK-BR-3 cells were seeded into a well in a 96-well plate and cultured overnight. The equal amount of Cy5 mRNA loaded pNB-LnP^200^ through pNB-LNP^2000^ was added into wells and incubated with SK-BR-3 cells for 30 min at 37 °C. Cells were fixed with 4% paraformaldehyde at 4 °C for 10 min followed by DAPI staining. Fluorescence images of cells were acquired.

### UV absorption spectra

The UV-Vis absorption spectra of pNB-LNP at different ratios were recorded using a UV-2550 UV-Vis spectrophotometer (Shimadzu, Japan). As a control, the spectra of a mixture without pNB were also measured.

### pNB anchor efficiency

pNB-anchored LNP were prepared as previously described. The Micro-BCA protein assay (ThermoFisher, 23235) was used to determine the membrane anchoring efficiency of pNB. A standard curve was generated following the manufacturer's instructions. In pNB-LNP samples, the ratio of pNB to a single LNP was set at 200:1, 500:1, 1000:1, 2000:1 and 4000:1. Subsequently, 1 mL of pNB-LNP was loaded into Amicon ultra centrifugal filters (300 kDa) containing 9 mL of PBS, and free pNB were separated by centrifuging the samples at 3,500 rcf for 30 min. This step was repeated twice to remove free pNB. The concentration of free pNB was then measured by Micro-BCA using the manufacturer's protocol after concentrated by Pierce™ Protein Concentrators PES, 3K (ThermoFisher, 88512). Based on NTA particle counts and the amount of anchored pNB, we calculated the average number of pNB molecules per LNP.

### Förster resonance energy transfer (FRET) analysis with NBD/Rhodamin

FRET experiments on pNB-LNP were conducted using a fluorometer (Spark, Tecan). For the NBD-PE/Rhodamine-PE donor/acceptor pair, the excitation wavelength was set at 460 nm, and emission spectra were collected from 500 to 650 nm. FRET was measured in pNB-LNP containing known donor- and acceptor-labeled vesicles concentrations. pNB-LNP samples were labeled with 1 mol% of both NBD-PE and Rhod-PE. To prepare pNB-LNP, stock ethanol solutions of the lipid mixture (450 SM102 : 60 DSPC : 290 DOPE : 200 cholesterol), NBD-PE, and Rhod-PE were combined to achieve the desired ratio (1000 µM lipid mix : 10 µM NBD-PE : 10 µM Rhod-PE). Subsequently, 0.015 and 0.054 µM pNB were added in PBS to 1 mL of pNB-LNP samples, respectively, following the previously described protocol. Similarly, LNP containing NBD-PE and Rhod-PE were prepared as controls for FRET analysis. Negative control LNP for pNB-LNP^200^ were prepared by mixing 1000.015 µM lipid mix, 10 µM NBD-PE, and 10 µM Rhod-PE, while those for pNB-LNP^4000^ were prepared with 1000.054 µM lipid mix, 10 µM NBD-PE, and 10 µM Rhod-PE. Additionally, donor-labeled vesicles (NBD-PE only) were prepared to calculate FRET efficiency (E) in the absence and presence of the acceptor, using the formula: *E*(%)= 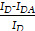
×100%, where *I_D_* is the donor intensity of samples containing only donor-labeled particles and I_DA_ is the donor intensity in samples with both donor- and -receptor-labeled particles.

### Laurdan assay

The membrane fluidity of pNB-LNP and LNP was measured with Laurdan. The polarization value (GP340) was calculated as GP340= 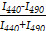
*,* where *I_440_* and *I_490_* are the emission intensities at 440 and 490 nm, respectively, of Laurdan excited at 340 nm. Measurement were conducted over a temperature range of 10 to 42 °C.

### Lysosome staining

Approximately, 2×10^5^ LNP were incubated with 5×10^3^ SK-BR-3 cells in a 96-well plate for 3 h at 37 °C followed by DAPI staining and lysosome staining (LysoView 594, Biotium) for 15 min. After thorough rinsing thrice, fluorescence images were acquired for qualitative analysis.

### Differential scanning calorimetry (DSC)

Approximately 50 µL of pNB-LNP samples were placed in aluminum pans and sealed with lids. A sample pan containing only saline was used as a reference. Samples were scanned over a temperature range of 10-60 °C at a heating rate of 10 °C/min.

### Circular dichroism (CD)

CD data was collected using a JASCO J-1500 spectropolarimeter (Spectra Manager II), equipped with a 1-cm optical path length cell at 25 °C. Measurements were performed for ~0.01 mM pNB, LNP, and pNB-LNP^2000^ in PBS.

### Luciferase assay

SK-BR-3 and MDA-MB-231 cells were seeded at a density of 3×10^4^ cells per mL in 96-well tissue culture plates. After 24 hours, the cells were treated with mRNA-pNB-LNP containing luciferase mRNA at concentrations ranging from 0 to 1,000 ng. Following an additional 24-hour incubation, luciferase activity was quantified using the Luciferase Assay (Promega), according to the manufacturer's instructions.

### mRNA transfection *in vitro* and binding assay

SK-BR-3 cells were cultured in DMEM (ThermoFisher, 11966025) supplemented with 10% fetal bovine serum (FBS, ThermoFisher, A5670701). After two days, the cells were seeded into 12-well plates at a density of 2×10^5^ cells per well and incubated in reduced serum media containing SARS-CoV-2 mRNA-pNB-LNP with 100 ng of mRNA. Following a two-day incubation, the cells were collected and lysed in Triton lysis buffer supplemented with a protease inhibitor cocktail. The supernatants were collected and stored at -20°C for further analysis. The concentration of SP was quantified using the SARS-CoV-2 SP sandwich ELISA kit (Invitrogen). For *in vitro* binding and microscopic analysis, Cy5-labeled mRNA-pNB-LNP at various ratios were incubated for 30 min and analyzed using flow cytometry and fluorescence microscopy. MDA-MB-231 cells served as a negative control. To assess cell viability, 0.5 mL of serum-free media containing the assay reagent was added to each well. After a 2-hour incubation at 37 °C, 100 μL of media was transferred to black-bottomed 96-well plates, and fluorescence signals were measured using a microplate reader (Spark, Tecan) at 540/590 nm. Data were expressed as percentages of fluorescence intensity relative to untreated controls.

### SP-antibody titration assay

The reactivity of ELISA against the expressed SP was evaluated. Plates were coated with SARS-CoV-2 proteins at 500 ng per well in phosphate-buffered saline (PBS) and incubated overnight at 4 °C. Following two washes with PBS, the wells were blocked using a 3% blotting-grade protein blocker (Bio-Rad) to prevent non-specific binding. Primary antibodies (Invivogen, CR3022) and spike antibodies (Sino Biological, 101290-T38) were introduced at concentrations varying from 1 to 10 μg/mL. Detection was conducted utilizing horseradish peroxidase (HRP)-conjugated donkey anti-human secondary antibodies (Jackson ImmunoResearch Laboratories, 709-006-149) at a dilution of 1:5,000. These secondary antibodies were pre-adsorbed to IgGs from various species to minimize non-specific interactions. Following a 10-minute incubation with 3,3',5,5'-tetramethylbenzidine (TMB) substrate, the reaction was halted by adding 50 μL of 2N sulfuric acid. Absorbance at 450 nm was recorded using a plate reader.

### Small angle X-ray scattering (SAXS)

mRNA-pNB-LNPs were prepared and loaded into quartz capillaries (Hilgenberg Glas) for SAXS analysis. Data was collected using a laboratory beamline (Xeuss 2.0 HR) equipped with a GeniX3D microfocus sealed tube beam source, operating at an X-ray wavelength of 1.54 Å with 50 kV and 0.6 mA power settings. Scattering data were recorded using a Dectris Pilatus3 R200K detector, with a sample-to-detector distance of approximately 1.2 m for the 2-pipe setup and 2.5 m for SAXS (3-pipe) data collection. Experiments were conducted at room temperature, with collimation achieved through silicon scatterless slits (0.5 and 0.3 mm). The collected two-dimensional scattering images were processed using Foxtrot 3.3.4 software (Xenocs), converting them into one-dimensional scattering data of intensity I(q) versus 2θ.

### Cryo-EM

Lacey carbon-coated 300-mesh copper grids (Electron Microscopy Sciences) were glow-discharged at 15 mA for 30 s using the PELCO easiGLOW glow discharge system (Ted Pella). A 3 µL aliquot of each sample was applied to a freshly glow-discharged grid for vitrification. The grids were blotted for 3 s under 95% relative humidity before being plunge-frozen in liquid ethane using a Vitrobot. Cryo-EM data was acquired at 300 kV using a Titan Krios microscope with a spherical aberration corrector.

### Immune killing *in vitro*

Peripheral blood mononuclear cells were obtained from healthy human volunteers (OriBiotech, T0028 and Z0229). CD3^+^ cells were isolated with a commercial kit (Stemcell, 17953). CD14^+^ cells were isolated via magnetic bead separation (Miltenyi, 130-050-201) followed by cultured in X-VIVO15 (Lonze, 04-418Q) serum-free medium supplemented with 20 ng/mL GM-CSF (Stemcell, 78190-C) and 30 ng/mL IL-4 (Stemcell, 78147.1) at 37 °C. SP-expressing HEK293T cells were cultured in DMEM (ThermoFisher, 11966025) supplemented with 10% FBS (ThermoFisher, A5670701) and 1 µg/mL puromycin (ThermoFisher, A1113802). On day 6, the experimental group received either 12.5 µg/mL or 25 µg/mL of SP (Acro Biosystems, S1N-C52Ha), while the control group received cytokines (Stemcell, 10989) only. Cells were cultured for an additional 48 hours. On day 7, 20 µg/ml Poly:IC and IFN-γ were added to mature DCs. On day 8, induced dendritic cells (DC) were harvested and subjected to flow cytometric analysis using antibodies against CD14 (Biolegend, 301813, 1:100), CD83 (Biolegend, 305305, 1:100), CD40 (Biolegend, 313013, 1:100), CD86 (Biolegend, 374205, 1:100), and CD80 (Biolegend, 375403, 1:100). SP-loaded DC were adjusted to a concentration of 1×10^5^ cells/mL, and 100 µL was added to each well of a 96-well plate. DC and CD3^+^ T cells were co-cultured at a 1:10 ratio for 5 days in RPMI 1640 (ThermoFisher, 12633012) supplemented with 10% FBS (ThermoFisher, A5670701), 5 ng/ml IL-7 (Stemcell, 78053.1), and 100 IU/mL IL-2 (Stemcell, 78036.1). After 3 days, the DC culture and SP loading procedure was repeated for secondary stimulation. Following a further 4 days of culture, co-cultured DC and CD3^+^ T cells were collected and counted. DC used for secondary stimulation were co-cultured with primarily stimulated CD3^+^ T cells at a 1:10 ratio for 48 hours. DC-CD3^+^ T cells were then co-cultured with SP-expressing HEK293T cells, MDA-MB-231 cells, or SK-BR-3 cells (1×10^4^ cells/well) at effector-to-target ratios of 1:1 and 5:1. Cytotoxicity was assessed at 6-, 24-, and 48-hours post-culture.

### Real-time cell analysis (RTCA)

SK-BR-3 cells with or without treatment with pNB-LNPs (2×10^4^ cells/well) were seeded in 96-well RTCA E-Plate (Agilent) and left to adhere for 18 h. On the day of the co-culture cytotoxicity experiments, activated SP-CD3^+^ T cells and naïve CD3^+^ T cells were added at an effector/target cell ratio of 0:1, 1:1, 5:1, and 10:1. An electrical current was applied to each co-culture. E-plates were read every 150 min for 24 h, and data were normalized to starting resistance. The normalized cell index was plotted as fold change against time. Once the co-culture incubation was finished, the supernatants from the RTCA plates and effector cell plate were then collected for IFN-γ analysis using DuoSet Human IFN-*γ* ELISA kit (DY285B, R&D Systems) following the manufacturer's protocol.

### Biodistribution *in vivo*

A subcutaneous SK-BR-3 breast tumor model was established for 14 days. NSG mice (n=3) were randomly divided into two groups and were intravenously injected with either pNB-LNP^2000^ or LNP-PEG, both encapsulating Cy5.5-labeled mRNA at a dose of 5×10^12^ LNPs per kg body mass. Mice were imaged using the IVI Spectrum system for up to 72 hours before being euthanized. Major organs and tumors were further collected and imaged.

### Immune killing *in vivo*

Six-week-old NSG mice were maintained in a barrier system, each in their own ventilated cage. Environmental conditions were carefully regulated: temperature was maintained between 20 and 26 °C, relative humidity ranged from 40 to 70%, a 12-hour light-dark cycle was enforced, and cage air was exchanged more than 50 times per hour. The mice were given unrestricted access to both a certified rodent diet and sterilized municipal tap water, which was provided in water bottles. Approximately 2×10^6^ SK-BR-3 cells in 50 μL PBS mixed with 50 μL Matrigel were inoculated subcutaneously into the flanks of NSG mice and allowed to grow until tumors reached ~150 mm^3^. The mice were then randomly divided into four groups. Mice received tail vein injections of SP mRNA-loaded pNB-LNP^2000^, LNP-PEG, or PBS at a dose of 2 mg mRNA/kg body mass. mRNA vaccine administration was repeated every five days for a total of six doses. Additionally, a single dose of ~1×10^7^ SP-CD3⁺ T cells per mouse was administered intravenously on day 0. Tumor volume was calculated every 3-4 days using the formula 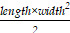, and body mass was measured concurrently. Mice that lost ~20% of their baseline body weight were considered to have reached a humane endpoint and were euthanized. On day 28, all remaining mice were euthanized for tumor and major organ collection.

### Statistical analysis

All data are presented as mean ± standard deviation (SD). Statistical comparisons between two groups were performed using Student's t-test, while comparisons among multiple groups were conducted using one-way analysis of variance (ANOVA) followed by Tukey's test, using GraphPad Prism 9.5.0. A *p*-value of <0.05 was considered statistically significant. Each experiment was repeated independently at least three times.

## Supplementary Material

Supplementary figures.
