# Supplementary material for: Tumor mRNA-lipid nanoparticles via chimeric nanobody-lipid co-assembly
Source: Theranostics. 2026 Jan 1;16(2):651–67. doi: 10.7150/thno.123633 (PMC12674939; doi:10.7150/thno.123633)
Supplement: Supplementary file 1 — Supplementary figures. [file thnov16p0651s1.pdf]

A

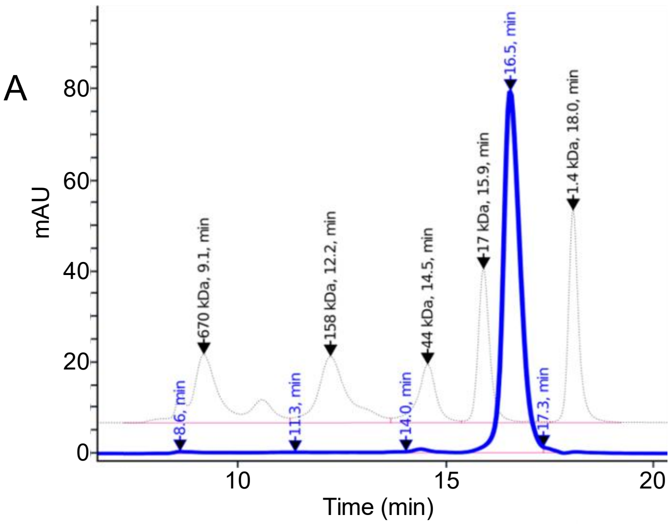

B

RT (min)	Height	Width (min)	Area	Area%
8.6	0.5	2.1	30.1	1
11.3	0.2	2.9	35.0	1
14.0	0.3	0.9	11.1	0
16.5	79.4	3.3	2315.4	96
17.3	1.2	0.4	15.7	1

C

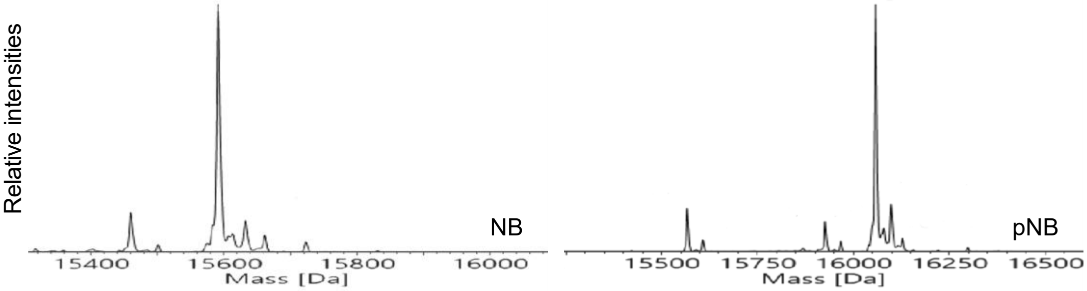

Figure S1. *Characterization of pNB*. (A) SEC-HPLC analysis of purified pNB and quantified purity. (B) Quantitative data. (C) Confirmation of conjugation of palmitic acids with MALDI-TOF-MS.

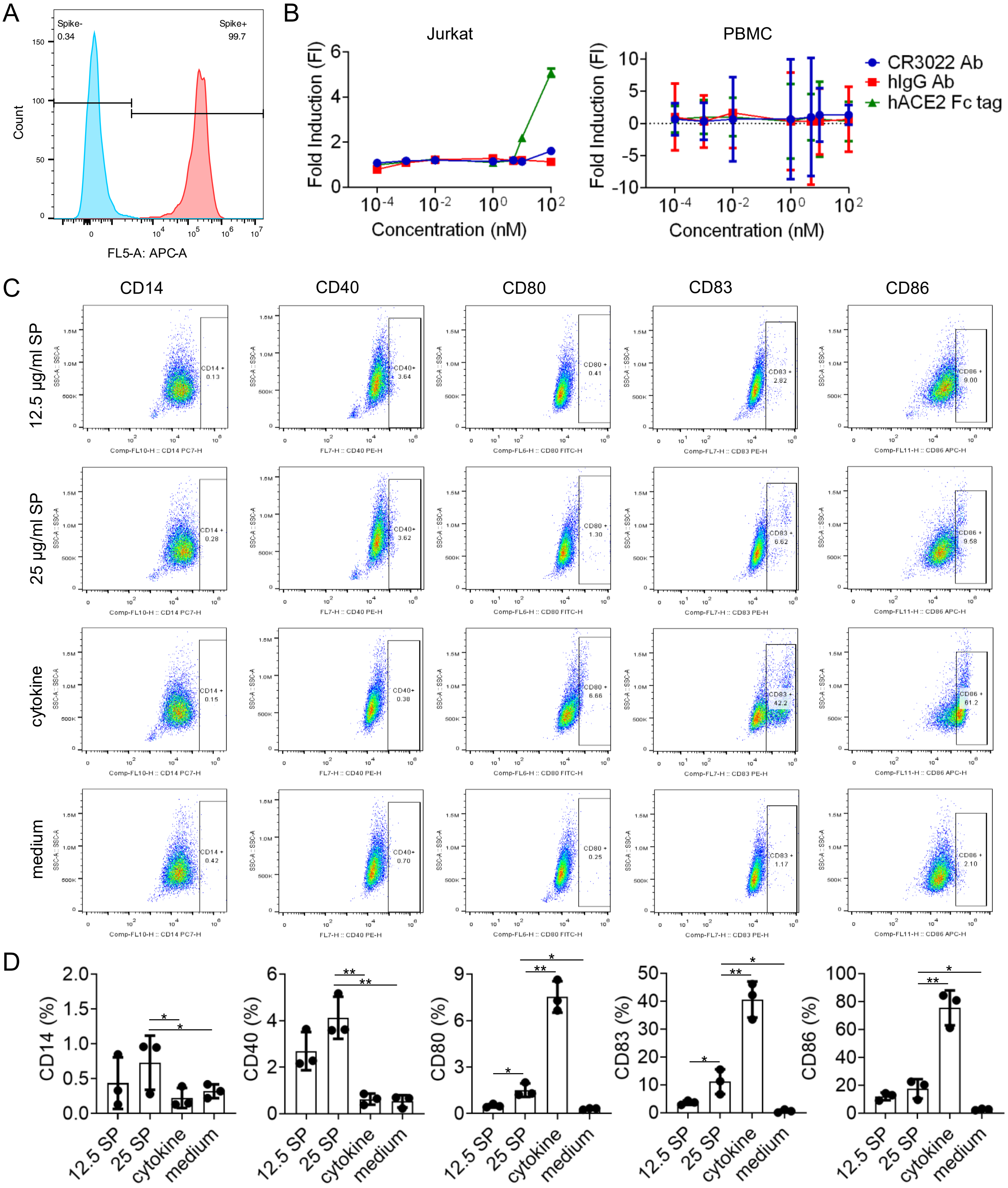

Figure S2. *Characterization of SP-expressing HEK293 cells, antibody-dependent cellular cytotoxicity, and optimization of SP dose for DCs.* (A) SP expression levels in experimental HEK293 cells. (B) Antibody-dependent cellular cytotoxicity using Jurkat cells and peripheral blood mononuclear cells. (C-D) Cytokines and medium were used as controls. Expression of CD14, CD40, CD80, CD83, and CD86 was assessed using flow cytometry, and the data were further quantified. Each data represents the Mean±SD of three replicates: *$p < 0.05$, **$p < 0.01$, and ***$p < 0.001$.

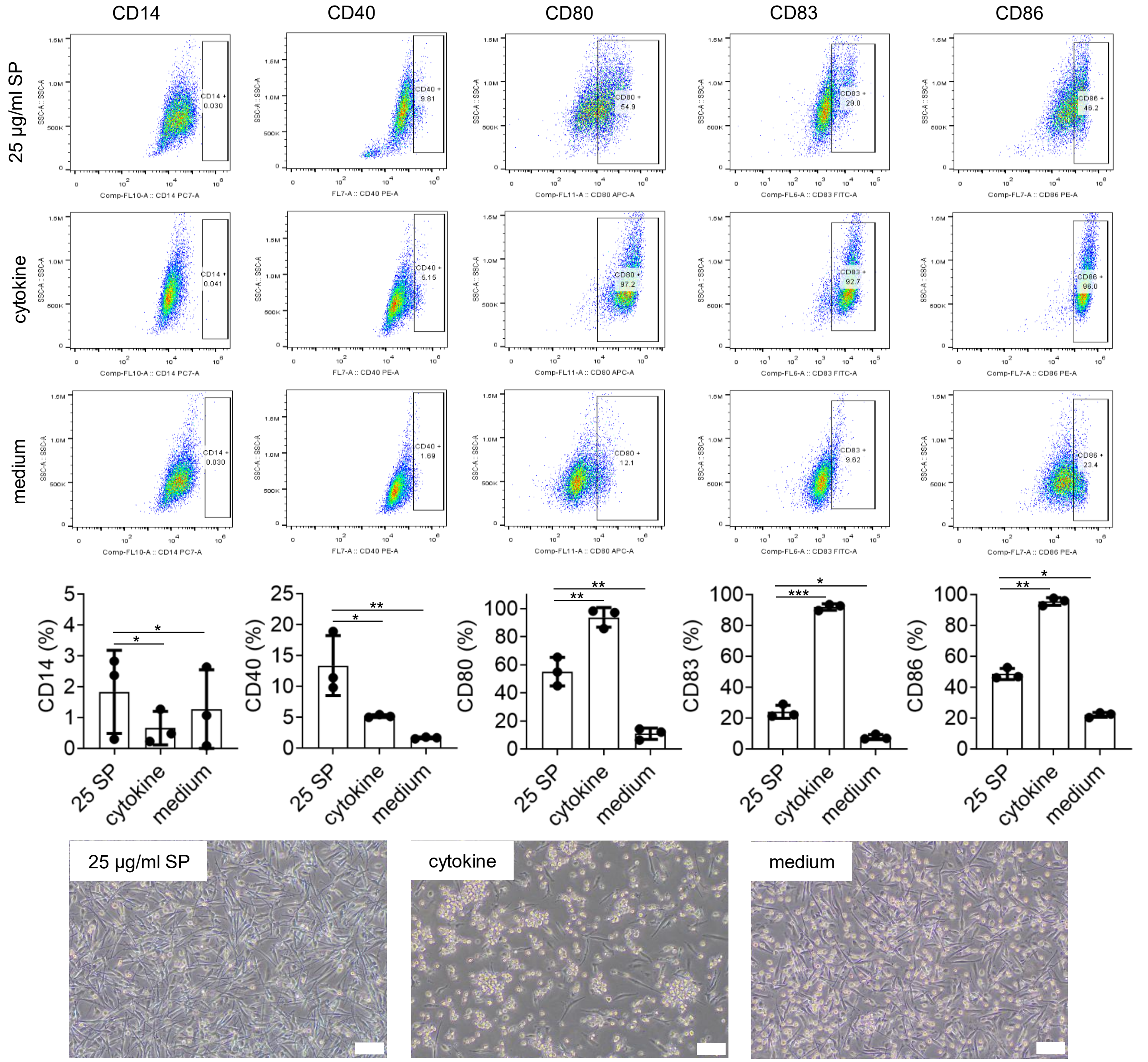

Figure S3. *Characterization of mature DCs.* Expression of typical makers was assessed using flow cytometry, and the data were further quantified. The morphology of mature DCs were observed under a light microscope. Each data represents the Mean±SD of three replicates: *p < 0.05, **p < 0.01, and ***p < 0.001.

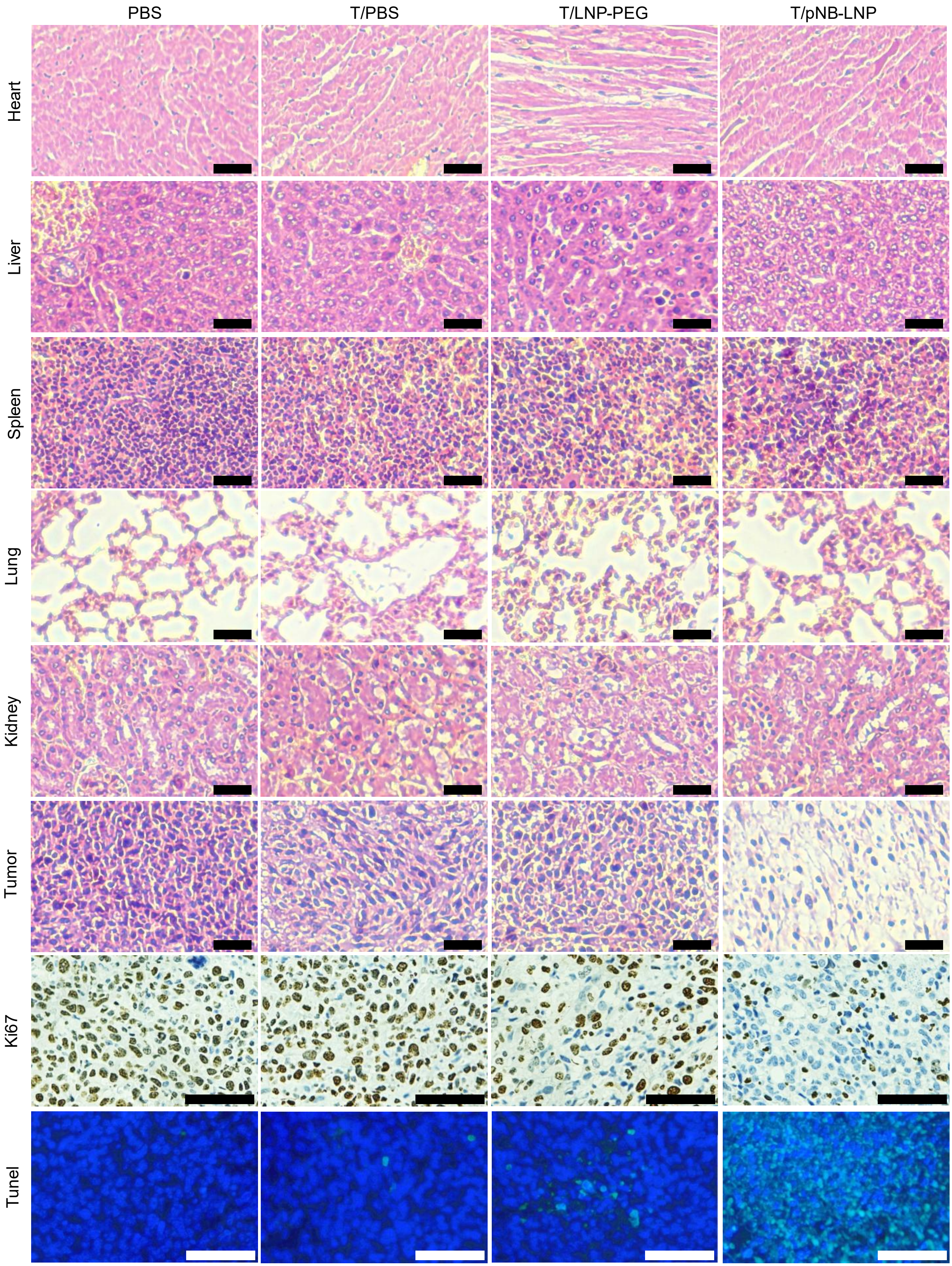

Figure S4. *Tumor treatment in vivo.* Representative images of H&E staining, Ki67 staining, and Tunel staining.
